# Notices of Some New Works

**Published:** 1839

**Authors:** 


### CIRCUMSPECTIVE REVIEW.

" Ore trahit quodcunque potest, atque addit acervo."

### Notices of some New Works.

Inaugural Dissertation on the Physiological Inferences to be deduced from the Structure of the Nervous System in the Invertebrated Classes of Animals. Submitted to the Medical Faculty of the University of Edinburgh, in conformity with the rules for graduation, by authority of the Very Rev. Principal Baird, and with the sanction of the Senatus Academicus. By *William B. Carpenter*, M.R.C.S., Late President of the Royal Medical and Physical Societies of Edinburgh, &c. and Candidate for the Degree of Doctor in Medicine. Edinburgh, John Carfrae, 8vo. pp. 84, 1839.

Mr. Carpenter is very favourably known for the "Principles of General and Comparative Physiology," a work which he has lately published, and which we had the pleasure of noticing.

A glance at the title will display the object of the work of Mr. Carpenter—of it's execution we can speak in terms of approbation—and it's results are summed up in the following "Conclusions," not of things proved, but of things probable. They shew the existing state of our knowledge and our ignorance on one of the most important subjects in Physiology—the nervous system. Aur author concludes :—

"I. That a nervous system, in the form of connected filaments with ganglia on certain parts of them, exists in all Animals (that is, in all beings endowed with any degree of sensibility and voluntary power), although its presence may not be detected by our means of observation.

II. That the actions most universally performed by a nervous system are those connected with the introduction of food into the digestive cavity.

III. That we have reason to regard this class of actions as everywhere independent of volition, and perhaps also of sensation;—the propulsion of food along the œsophagus in man being of this character.

IV. That, for the performance of any action of this nature, a nervous circle is requisite, consisting of an *afferent* nerve on the peripheral extremities of which an impression is made;—a ganglionic centre, where the white fibres of which that nerve consists terminate in grey matter, and those of the efferent nerve originate in like manner;—and an *efferent* trunk conducting to the contractile structure the motor impulse, which originates in some change in the relation between the grey and white matter.

V. That such actions may be regarded as the simplest of those which the nervous system performs, and most resemble the examples of contraction produced by the irritation of distant organs in Plants, (where an *impression* is mechanically conveyed by the circulating system,) of any which the Animal Kingdom affords.

VI. That in the lowest Animals such actions constitute nearly the entire function of the Nervous System; the amount of those involving sensation and volition being very small.

VII. That, as we ascend the scale, the evidence of the participation of true sensation in the actions necessary for the acquirement of food, as shown by the development of special sensory organs, is much greater; but that the movements *immediately* concerned with the introduction of food into the stomach remain under the control of a separate system of nerves and ganglia, to the action of which the influence of the cephalic ganglia,—the *special* if not the *only* seat of sensibility and volition,—is not essential.

VIII. That, in like manner, the active movements of respiration are controlled by a separate system of nerves and ganglia, and are not dependent upon that of sensation and volition, though capable of being influenced by it.

IX. That the centres of these systems are brought into closer structural relation with that of the sensori-volitional system as we ascend the scale of Invertebrated Animals; until they at last apparently become a part of it, as in Vertebrata, where, however, they still remain really separate, and may be artificially insulated.

X. That, whilst the actions of these systems are in the lower tribes almost entirely of a simply—reflex character, we find them, as we ascend, gradually becoming subordinated to the will; and that this is effected by the mixture of fibres proceeding directly from the cephalic ganglia with those arising from their own centres.

XI. That the locomotive organs, in like manner, have their own centres of reflex action, which are independent of the influence of volition, perhaps also of sensation.

XII. That the influence of the will is conveyed to them by separate nervous fibres, proceeding from the cephalic ganglia; and that similar fibres probably convey to the cephalic ganglia the impressions destined to produce sensations.

XIII. That the stomato-gastric, respiratory, and locomotive centres are all united in the spinal cord of Vertebrata, where they form one continuous ganglionic mass; and that the nerves connected with all these also receive fibres derived immediately from the cephalic ganglia.

XIV. That whenever peculiar consentaneousness of action is required between different organs, their ganglionic centres are united more or less closely; and that the trunks themselves are generally connected by bands of communication.

XV. That the sympathetic system does not exist in the lowest classes in a distinct form;—that the nervous system of the Invertebrata, taken as a whole, bears no analogy with it;—that, as the divisions of this become more specialised, some appearance of a separate sympathetic presents itself;—but that this is never so distinct as in Vertebrata.

XVI. Hence it may be inferred that, as the sympathetic system is *not* developed in proportion to the predominant activity of the functions of organic life, but in proportion to the development of the higher division of the nervous system, its office is not to 'preside over' the former, but to bring them into relation with the latter; so that the actions of the organs of Vegetative Life are not dependent upon it, but influenced by it in accordance with the operations of the system of Animal Life."

